# Engineering Diffusion-Controlled Drug Delivery Systems to Achieve a Desired Drug Release Profile: Mathematical Modelling and Optimisation

**DOI:** 10.1007/s11095-026-04149-6

**Published:** 2026-08-03

**Authors:** Daniele Peri, Elliot J. Carr, Giuseppe Pontrelli, Sean McGinty

**Affiliations:** 1 Istituto per le Applicazioni del Calcolo, CNR, Rome, Italy; 2 School of Mathematical Sciences, Queensland University of Technology, Brisbane, Australia; 3 Division of Biomedical Engineering, University of Glasgow, Glasgow, UK; 4 Glasgow Computational Engineering Centre, University of Glasgow, Glasgow, UK

**Keywords:** Drug delivery, Optimisation, Mathematical modelling, Zero-order release, Functionally graded material

## Abstract

**Objective:**

Drug delivery devices offer a promising strategy for the localized treatment of disease while minimising systemic side effects. However, designing diffusion-controlled systems to achieve a prescribed drug release profile remains a major challenge due to their inherently time-varying release rates. This study aims to develop a mathematical framework to enable the rational design of such systems to achieve desired therapeutic release profiles.

**Methods:**

We develop a continuum-scale mathematical model describing diffusion-controlled drug release from a spherical microcapsule composed of a functionally graded polymer with spatially varying diffusivity and non-uniform initial drug distribution. These spatial design variables are parameterised using Bézier curves and identified via an inverse optimisation procedure based on a hybrid imperialist competitive algorithm. The framework is applied to two inverse design problems: (i) achieving near constant (zero-order) drug release over a specified time window, and (ii) reproducing prescribed release profiles defined by target checkpoints.

**Results:**

The results show that a constant release rate can be achieved by tailoring the initial drug distribution while selecting an appropriate constant diffusivity. In contrast, more general release profiles require simultaneous optimisation of both spatially varying diffusivity and initial drug loading.

**Conclusions:**

The proposed framework demonstrates how mathematical modelling and optimisation can enable the rational design of diffusion-controlled drug delivery systems capable of producing tailored therapeutic release behaviours.

**Fig. 1:**
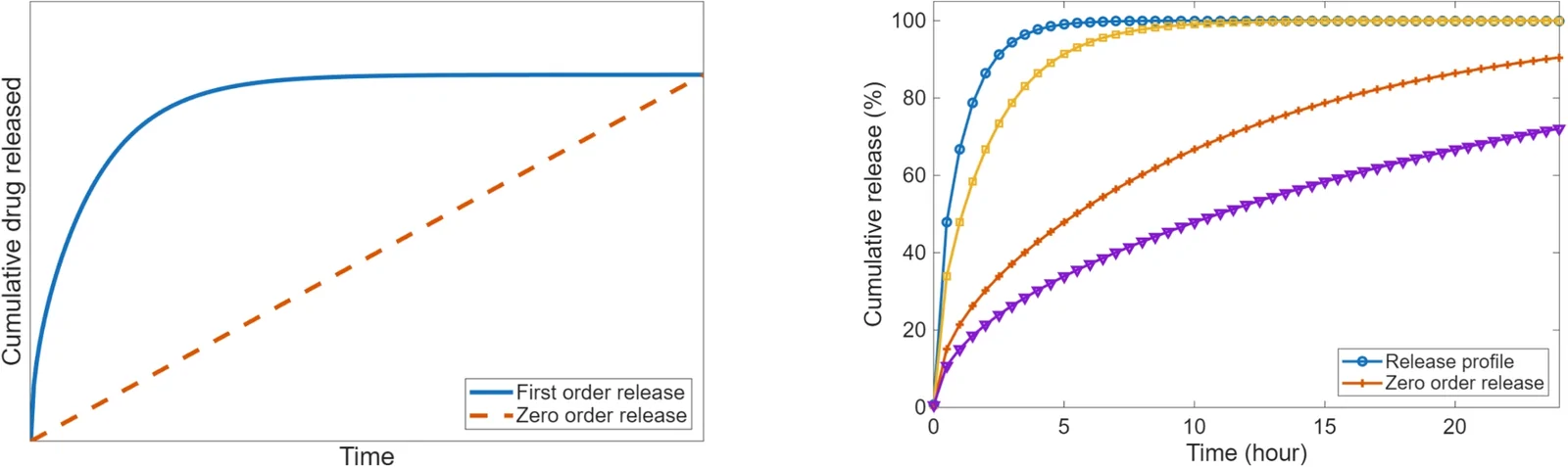
Left: Comparison between first order and zero order release kinetics. Right: examples of typical drug release profiles from diffusion-based DDS

## Introduction

The design of drug delivery systems (DDS) capable of achieving a desired drug release profile has been the subject of extensive research over several decades [1–3]. This interest is motivated by the critical role that *controlled release* plays in modern therapeutics, where the rate of drug delivery can strongly influence both treatment efficacy and patient safety. As an alternative to more conventional administration routes that often produce large variations in drug concentration (e.g. systemic delivery), DDS are increasingly being engineered to provide more predictable and controllable release behaviour. This is particularly important for drugs with narrow therapeutic windows, or significant dose-dependent side effects, where maintaining drug concentrations within a desired range is essential [4]. A wide range of DDS have been developed to address these challenges. These may be broadly classified by their dominant release mechanism, e.g. diffusion-controlled, dissolution-controlled and swelling-controlled. Examples include tablets in the form of polymeric matrices [5], reservoir devices with rate-controlling membranes [6], microspheres (capsules) and more sophisticated stimuli-triggered systems [7, 8] that respond to environmental changes such as in pH or temperature. Each of these DDS offers distinct benefits and limitations depending on the drug properties, route of administration, and desired release profile. However, despite this diversity, diffusion-controlled systems remain among the most widely studied and frequently employed controlled release strategies [9, 10].

Diffusion-controlled DDS have attracted sustained attention largely because of their simplicity, predictability, and manufacturability [11]. In such systems, drug release is governed primarily by the diffusion of dissolved drug molecules through a polymeric matrix or across a membrane barrier into the surrounding environment. These systems are attractive because they can often be described using well established transport equations, giving rise to mathematically tractable models that support rational design and optimisation [9]. In addition, diffusion-controlled devices are generally straightforward to fabricate using scalable methods already familiar to the pharmaceutical industry. This combination of theoretical simplicity and practical feasibility has made diffusion-based delivery systems a foundational topic in both academia and industry. Nevertheless, diffusion-controlled systems have their own set of limitations. In particular, they sometimes prove restrictive in terms of the range of drug release profiles that can be achieved. A common feature of these systems is an initially high release rate followed by a progressive reduction as diffusion pathways lengthen and drug concentration gradients diminish as a result of drug being released, giving rise to first order release kinetics (Fig. 1). In many cases, diffusion-controlled devices also exhibit burst release, where a substantial fraction of the loaded drug is released rapidly at early times [4]. This burst is often associated with drug located near or on the device surface, or initial steep concentration gradients. Such behaviour can be advantageous when an immediate therapeutic effect is desired but it may also be undesirable if it causes toxic concentrations or reduces the longevity of DDS.

More generally, diffusion-controlled DDS tend to produce release profiles whereby the drug release rate varies non-linearly with time. While this may match certain therapeutic needs, it can be incompatible with applications requiring uniform drug dosing. The pharmaceutical industry often values constant (zero-order) drug release (Fig. 1), where the delivery rate remains steady over extended periods [12, 13]. This is appealing because it enables the sustenance of drug concentrations within a desirable therapeutic range, minimising peaks and troughs that might otherwise cause side effects or reduce efficacy. However, the intrinsic physics of diffusion in homogeneous media makes constant release difficult to obtain without additional mechanisms such as moving boundaries, swelling fronts, or time-varying transport properties [14, 15]. At the same time, it is important to recognise that constant release is not universally optimal. In certain clinical contexts, a particular drug release profile is desired, and this may not (and often will not) follow first-order kinetics. Therapeutic goals may require pulsed, delayed, ramped delivery, multi-phase dosing, or personalised time-varying profiles aligned with disease progression. For example, anti-inflammatory drugs might benefit from delayed onset, hormones may require rhythmic delivery, and oncology treatments might involve carefully structured dosing schedules to balance toxicity and efficacy. Therefore, the ability to engineer diffusion-controlled systems to realise a broader class of release behaviours beyond the standard diffusion-driven decay remains an important and unresolved challenge.

This work addresses this challenge by focusing on diffusion-controlled release from a spherical capsule geometry. Spherical capsules and microscale particles are of particular interest because they are widely used in pharmaceutical formulations (including microcapsules and microparticles), have high surface-area-to-volume ratios, and often admit relatively clean mathematical descriptions [16]. The central premise of this study is that, while diffusion-controlled systems are often seen as restrictive, significant control over release behaviour may still be achieved through rational manipulation of system properties. Specifically, rather than introducing additional release mechanisms, we consider whether desired drug release profiles can be obtained by varying only: (i) the initial spatial distribution of drug within the capsule, and/or (ii) a spatially varying diffusivity inside the device. These modifications preserve the diffusion-controlled nature of the system while introducing additional design degrees of freedom that may enable more sophisticated release patterns [17]. Importantly, such modifications may be realised through the use of functionally graded materials (FGM), a special class of materials that enable smoothly varying spatial properties, yet remain under-exploited in the context of drug delivery [18–21, 26]. Within this framework, we consider the following question: How can we engineer a diffusion-controlled drug delivery system to achieve either (1) constant drug release (zero-order kinetics), or (2) a generic target drug release profile specified in advance? To answer this question, we propose a combination of mathematical modelling and optimisation for controlling diffusion-driven release by design. The emphasis is not merely on characterising release profiles, but on establishing an inverse design capability: given a desired release behaviour, determine how the capsule properties and initial conditions should be engineered to achieve it. This approach aims to expand the functional scope of diffusion-controlled systems, supporting both industrial objectives such as constant release and more specialised therapeutic needs where tailored profiles are required.

## Methodology

### Mathematical Model of Drug Release

**Fig. 2:**
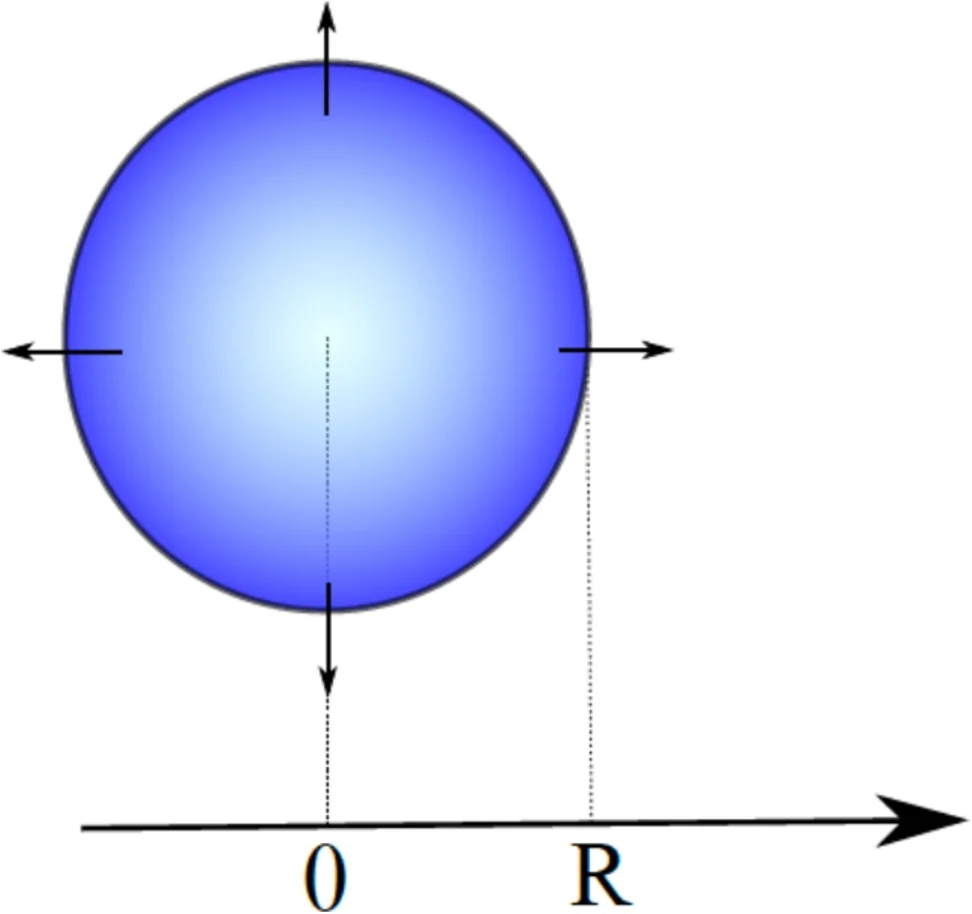
Schematic representation of a sphere consisting of an FGM releasing drug. The gradient of colour signifies smoothly varying material properties and/or initial non-uniform concentration

We consider a drug-loaded polymeric microsphere Ω in contact with a release medium. We suppose that diffusion is the primary mechanism of drug transport and that the microsphere is comprised of an FGM, possessing a spatially-varying drug diffusion coefficient $D({\textbf {x}})$ and possible non-uniform initial drug loading $c_0({\textbf {x}}$) (Fig. 2). The rate of drug release is then governed by a combination of the spatial distribution of drug diffusivity, initial concentration and microsphere radius (*R*), via the diffusion equation:

2.1 $$\begin{aligned} \frac{\partial {c}}{\partial t} = \nabla \cdot \left( D({\textbf {x}}) \nabla c \right) , \qquad {\textbf {x}} \in \Omega , \quad t \in [0, T], \end{aligned}$$

where $c({\textbf {x}},t)$ is the volume-averaged concentration. Assuming radial symmetry, we may reduce the problem to one-dimension. The model of drug release is then given by:

2.2 $$\begin{aligned}&\frac{\partial {c}}{\partial t} = {1 \over r^2}{\partial \over \partial r} \left( r^2 D(r) {\partial c \over \partial r} \right) , \qquad r \in [0, R], \quad t \in [0, T], \end{aligned}$$

2.3 $$\begin{aligned}&c(r,0) = c_0(r), \qquad r \in [0, R], \end{aligned}$$

2.4 $$\begin{aligned}&{\partial c \over \partial r }(0,t) = 0, \qquad t \in [0, T], \end{aligned}$$

2.5 $$\begin{aligned}&c(R,t)=0, \qquad t \in [0, T]. \end{aligned}$$

Equation (2.4) expresses the requirement of symmetry/finiteness at the center of the sphere, and Eq. (2.5) describes the release medium as a perfect sink, as is common in the literature [9]. We assume that the initial concentration $c_0(r)$ and diffusivity function *D*(*r*) are constrained to lie with the ranges $0 \le c_0(r) \le C_{\text {max}}$ and $[D_{\text {min}}, D_{\text {max}}]$, respectively. $D_{\text {min}}, D_{\text {max}}, C_{\text {max}}$ are arbitrary model constants, and their values are related to the specific problem and to manufacturing constraints.

Through the following change of variables:

2.6 $$\begin{aligned}&r \rightarrow {r \over R}, \qquad t \rightarrow {D_{\text {max}}t \over R^2}, \qquad c(r,t) \rightarrow {c(r,t) \over C_0},\end{aligned}$$

2.7 $$\begin{aligned}&c_{0}(r) \rightarrow \frac{c_0(r)}{C^*_{0}},\qquad T \rightarrow { D_{\text {max}}T \over R^2} \qquad D(r) \rightarrow { D(r) \over D_{\text {max}}}, \end{aligned}$$

with $C_0$ a reference value, the diffusion model (2.2)–(2.5) can be put in a non-dimensional form:

2.8 $$\begin{aligned}&\frac{\partial c}{\partial t} = {1 \over r^2}{\partial \over \partial r} \left( r^2 D(r) {\partial c \over \partial r} \right) , \qquad r \in [0, 1], \quad t \in [0, T], \end{aligned}$$

2.9 $$\begin{aligned}&c(r,0) = c_{0}(r), \qquad r \in [0, 1], \end{aligned}$$

2.10 $$\begin{aligned}&{\partial c \over \partial r }(0,t ) = 0, \qquad t \in [0, T], \end{aligned}$$

2.11 $$\begin{aligned}&c(1,t)=0 , \qquad t \in [0, T]. \end{aligned}$$

The above FGM problem has been solved in [18, 19] for a variety of choices of the diffusivity *D*(*r*). The drug fractional release profile, *P*(*t*), the cumulative fraction of mass released over time, is a key quantity measured by experimentalists to explore how changes in capsule design impact the rate of drug release. This may be calculated from the model via

2.12 $$\begin{aligned} P(t)= 1 - {M(t) \over M(0)}, \quad t \in [0,T], \end{aligned}$$

where *M*(*t*) is the time-varying mass of drug in the FGM:

$$\begin{aligned} M(t) = 4 \pi \int _0^1 r^2 c(r,t) dr, \end{aligned}$$

2.13 $$\begin{aligned} M_0 = M(0)= 4 \pi \int _0^1 r^2 c_0(r) dr. \end{aligned}$$

The mass flux (that defines the release rate) at the sphere surface is:

2.14 $$\begin{aligned} Q(t)= 4\pi \left. \left( -D(r) r^2 {\partial c \over \partial r}(r,t) \right) \right| _{r=1} \end{aligned}$$

and an alternative form of Eq. 2.12 is:

2.15 $$\begin{aligned} P(t)= {\displaystyle \int _0^t Q(s) ds \over M(0)} . \end{aligned}$$

Equations (2.8–2.11) were solved using the finite difference method, with the forms of *D*(*r*) and $c_0(r)$ as specified in Section “The Optimization Problem”. We approximate the second order spatial derivatives with a central difference scheme and the time derivative with a forward Euler discretization. The spatial interval [0, 1] is discretized uniformly by $\Delta r=1/K, \, K=250$ and the time is discretized adaptively with a time step Δt chosen such that $\displaystyle \max _i \, D(r_i) \, \Delta t/ \Delta r^2 = 0.5$ (CFL condition).

### The Optimization Problem

Our goal is to inversely estimate the drug coating properties *D*(*r*) and/or $c_0(r)$ to achieve a desired drug release profile *P*(*t*). Since *P*(*t*) is governed by the spatial distribution of *D*(*r*) and $c_0(r)$ through the underlying transport model (see Eqs. (2.14) and (2.15)), we aim to identify the optimal distributions *D*(*r*) and $c_0(r)$ at each grid node. To this end, these functions are represented in parametric form, thereby reducing the problem to the determination of a finite set of parameters, which serve as the decision variables in the resulting optimization problem. More specifically, we have chosen a pair of Bézier curves [22, 23] to represent *D*(*r*) and $c_0(r)$. This approach is advantageous because a complex curve can be defined by a relatively small number of control points, far fewer than the number of points in the calculation grid. This ensures smooth, regular functional forms for *D*(*r*) and $c_0(r)$, which are more practical from a manufacturing standpoint. We consider two Bézier curves of order J=10, each constructed using J+1 control points (CPs): $X_1, X_2,\ldots ,X_{J+1}$, for a total of 2J+2 unknown parameters. The spacing of CPs is not necessarily uniform; we allow for the *thickening* or *thinning* of points at the boundaries if such a distribution proves more favorable. This flexibility is managed by two additional parameters for each curve, so that the total number is M=2J+6, if both *D*(*r*) and $c_0(r)$ are subject to optimization. Each set of optimization variables (the locations of the Bézier CPs) defines a specific configuration for *D*(*r*) and $c_0(r)$. By solving the *direct problem* for a given configuration, we obtain a specific drug release curve. The quality of this solution is determined by the instantaneous deviation of the current release curve from the target profile. The objective function is defined as the average absolute deviation between these two curves. The optimization algorithm then searches the design space to minimize this objective function.

The optimization is carried out using a Hybrid Imperialist Competitive Algorithm (Hybrid-ICA), which is a global method proposed in [24] and improved in [25] based on multiple interacting agents. The algorithm uses names inspired by imperialist strategies, but in practice it works by spreading many possible solutions across the entire design space. It does not need an initial guess; only the allowed range of each design variable must be defined. These possible solutions (called agents) are divided into groups, called empires. In each empire, the best solution becomes the leader (the imperialist). The other agents in the group adjust their positions by moving around the leader, trying to improve their performance. The size of each empire is chosen based on the number of design variables, so the method can adapt to problems of different complexity. If an agent finds a better solution than the current leader, it becomes the new imperialist. The size of each empire changes over time depending on how well its members perform. At each step, the weakest agent from the least successful empire is moved to the most successful one, helping stronger empires grow. The “hybrid” feature means that if the global search does not improve the best solution, a local search method is activated. This allows the global and local strategies to work together. The process continues until all agents merge into a single empire and its size becomes small enough, or until a maximum number of evaluations is reached. For further details, see [24] and [25].

**Table 1:** Constraints on *D*(*r*) and $c_0(r)$ for the optimization problem

Constraint on *D*(*r*)	Constraint on $c_0(r)$
D1: $\quad D_{\text {min}}\le D(r) \!\le \! 1$	C1: $0 \le c_0(r) \le C_{\text {max}}$
D2: 1 $\searrow D_{\text {min}}$	C2: $\quad 4 \pi \displaystyle \int _0^1 r^2 c_0(r) dr \!=\! M(0) \!=\!$ const.
D3: $\displaystyle {dD \over dr} (0) =0$	C3: $\displaystyle {dc_0 \over dr} (0) =0$
D4: $\displaystyle {d^2D \over dr^2} (0) =0$	C4: $\displaystyle {d^2c_0 \over dr^2} (0) =0$

**Table 2:** Dimensional and nondimensional parameters of the problem. While microspheres have typical radii in the range 1-1000 μm, the lower end of this range is chosen, following [18]. The values of $D_{\text {max}}$ and $D_{\text {min}}$ cover a typical range of drug diffusion coefficients in durable polymers. The maximum initial concentration, $C_{\text {max}}$, and reference concentration, $C_{0}^*$, were chosen arbitrarily (but within a physically meaningful range), since the primary quantity of interest, the fractional drug release *P*(*t*), depends only on the ratio *M*(*t*)/*M*(0). As both *M*(*t*) and *M*(0) scale linearly with concentration, the choice of concentration value does not affect the predicted release profile

Parameter	Description	Value [dim.]	Value [non dim.]	Reference
*R*	Radius	$10^{-4}\,\text {cm}$	1	[18]
$D_{\text {min}}$	Minimum diffusivity	$10^{-13}\,\text {cm}^2/\text {s}$	$10^{-2}$	[18, 27, 28]
$D_{\text {max}}$	Maximum diffusivity	$10^{-11}\,\text {cm}^2/\text {s}$	1	[27, 28]
$C_{\text {max}}$	Max initial concentration	$0.4 \,\text {g}/\text {cm}^3$	10	This study
$C_{0}^*$	Reference concentration	$0.04 \,\text {g}/\text {cm}^3$	1	This study
$M_0$	Initial mass	$\frac{4}{3}\pi \, 0.04 \cdot 10^{-12} \, \text {g}$	$\frac{4}{3}\pi$	Calculated

In our problem, in order to obtain *D*(*r*) and $c_0(r)$ that are feasible from a physical and manufacturing standpoint, we restrict the search by imposing physical, geometric and manufacturing based constraints (Table 1). Specifically, *D*(*r*) lies in a finite range dictated by material limitations (D1), and is a strictly decreasing function (D2). Constraints D3 and C3 impose a natural symmetry condition at the centre of the sphere. Finally, flatness at r=0 is imposed by requiring the second derivatives to vanish there (conditions D4 and C4). Constraint C2 ensures that the initial mass of drug loaded on the sphere is fixed, while C1 imposes a maximum concentration value, which is related to manufacturing constraints.

## Results and Discussion

In this section we apply this methodology to two specific inverse problems of interest, with the parameters based on typical values in the literature as presented in Table 2.

### Targeting a Constant Release Rate

A useful (and simple) indicator of the release properties is the release rate *Q*(*t*), given by the slope of the release curve. While it may be desirable to attain some constant release rate, $Q^*$, in practice this is only achievable over a finite time period. Thus, we target a constant release rate over a defined time window, say $0<t<t^*$. For the purposes of this study, we fix $Q^*=0.2$ and $t^*=2.5$, although clearly the desired values could vary substantially depending on the specific drug delivery application. Thus, our goal is to minimize the following objective function

3.1 $$\begin{aligned} \mathfrak {F} (X_1, X_2,...,X_M) \!=\! \!\sqrt{\frac{\displaystyle \sum _{i=1}^{N^*} \left( Q(t_i) \!-\! Q^* \right) ^2}{N^*}} \quad \!\!\text{ in } \, [0,t^*], \end{aligned}$$

where $t_i, i=1...N^*$ are the discrete time points in $[0,t^*]$ and $X_1, X_2,...,X_M$ are the CPs defined in Section “The Optimization Problem”.

**Fig. 3:**
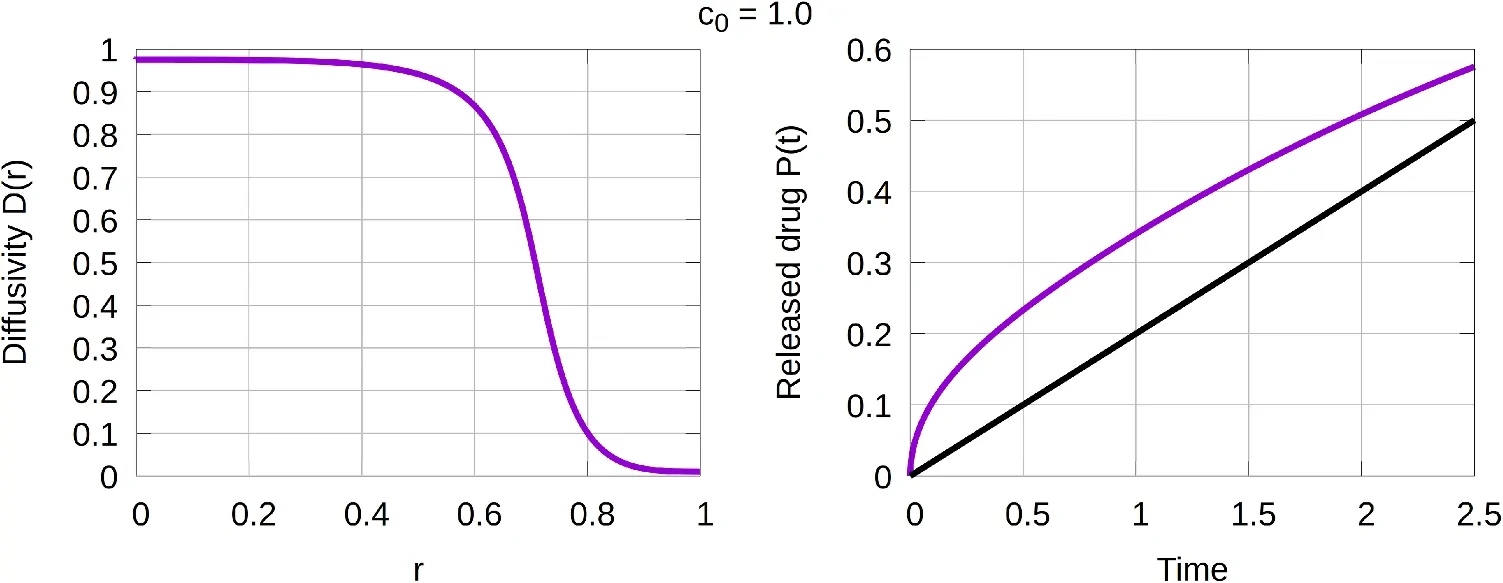
Initial attempt at optimization of the nondimensional diffusivity function *D*(*r*) for a constant nondimensional initial concentration of $$c_0=1$$ ($$ Q^*=0.2, t^*=2.5$$). Left: The optimal nondimensional diffusivity function. Right: Comparison between release profile obtained with optimal diffusivity function (purple) and the desired constant release rate (black)

We initially performed an optimisation over *D*(*r*) while keeping constant $c_0=1$. However, we were not able to obtain the desired $Q^*$ for $0<t<t^*$ (See Fig. 3) indicating that a spatially-varying diffusivity alone is insufficient to obtain a constant release rate for the desired time.

**Fig. 4:**
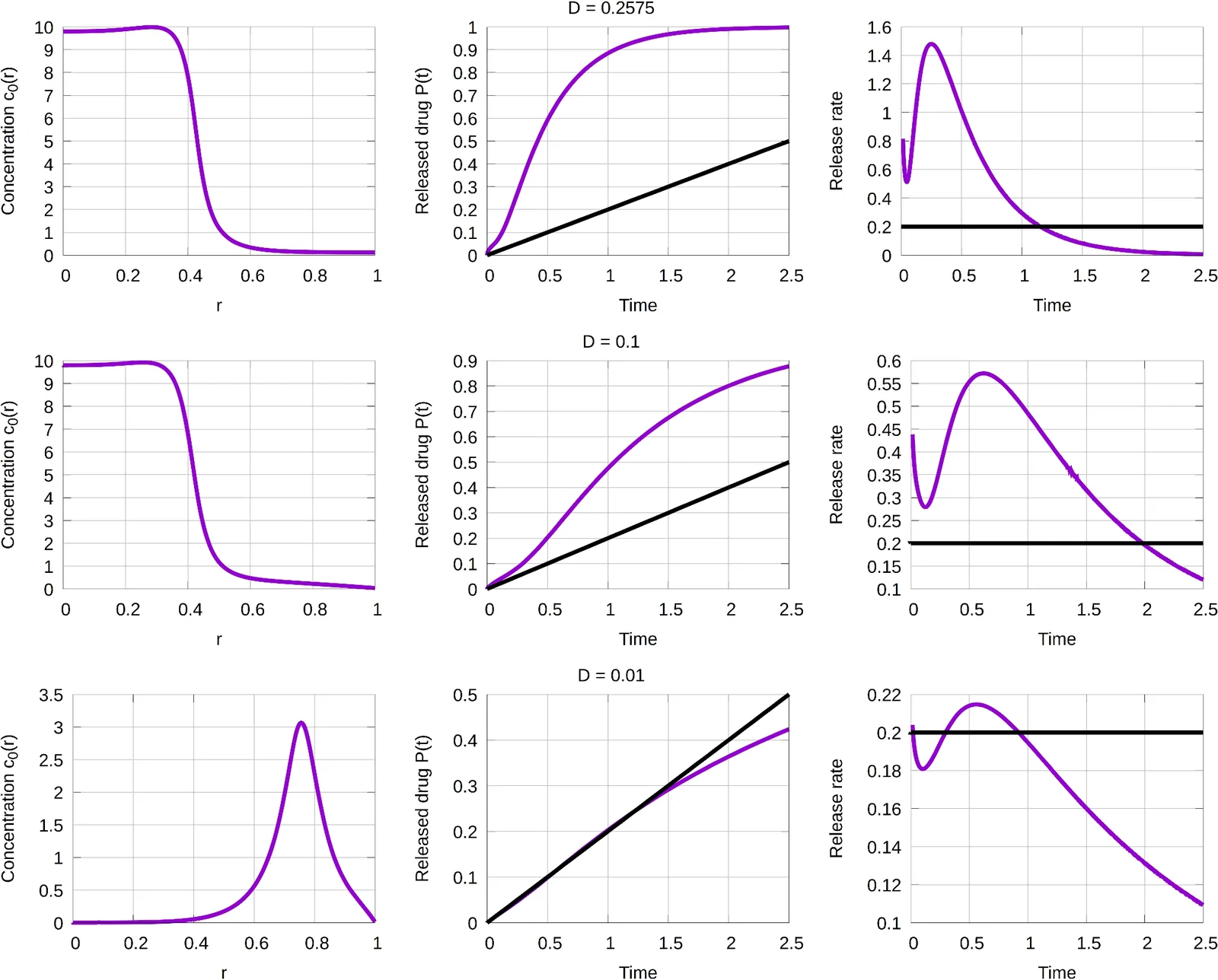
Optimization of the initial nondimensional concentration $c_0$ targeting $Q^*=0.2, t^*=2.5$ for three different constant diffusivity values (Top row: D=0.2575, Middle row: D=0.1, Bottom row: D=0.01). Each row displays: Left: nondimensional initial concentration profile, middle: drug release profile, right: rate of drug release. The black line indicates the target constant release rate

**Fig. 5:**
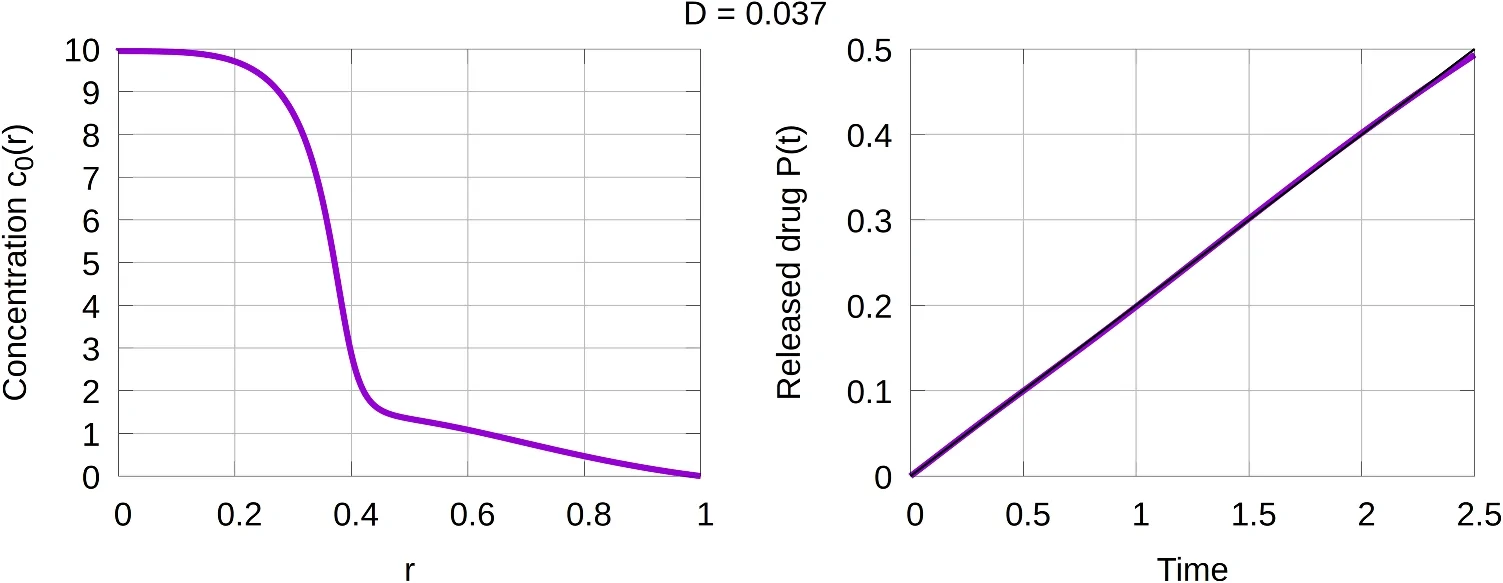
Simultaneous optimization of $$c_0(r)$$ and constant *D* (for $$ Q^*=0.2, t^*=2.5$$)

**Fig. 6:**
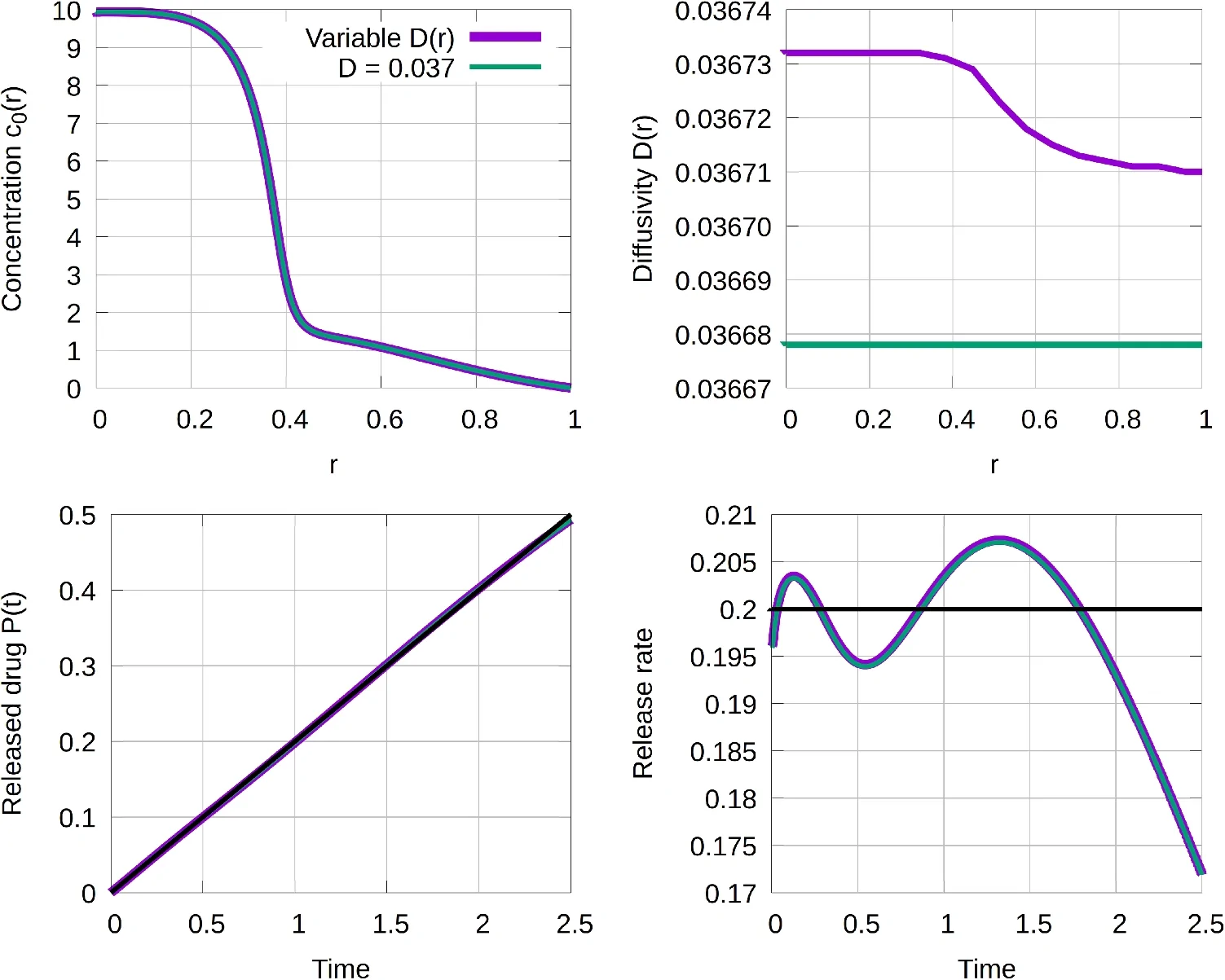
Comparison between optimisation over $c_0(r)$ performed with constant D=0.037 and variable *D*(*r*). While the spatial diffusivity profiles are very slightly different (Top Right), a perfect match is observed in the optimal $c_0(r)$ (Top Left), release profile (Lower Left) and release rate (Lower Right). Note that the scale in the Top Right plot has been chosen to emphasise the differences, although the values are clearly very similar

Next we performed the optimization over $c_0(r)$ assuming a constant diffusivity. As an initial step, we selected three different constant values for *D* and found that we were able to obtain the desired $Q^*$ for $0<t<t^*$ (See Fig. 4) by choosing an appropriate constant value of *D*. This confirms that it is sufficient to optimise over $c_0(r)$, provided *D* constant is chosen carefully. The results obtained by varying the value of spatially-constant *D* are somewhat intuitive: since *D* controls the timescale of the drug release, lower values reduce the release rate and prolong the initially linear rate of release. A further optimization over both $c_0(r)$ and constant *D*, yielded an optimal value of D=0.037 (See Fig. 5). A simultaneous optimisation over $c_0(r)$ and *D*(*r*) does not discernibly improve the results (See Fig. 6). Thus, we observe that optimization over $c_0(r)$ and *D* constant is sufficient to obtain the desired release profile.

**Fig. 7:**
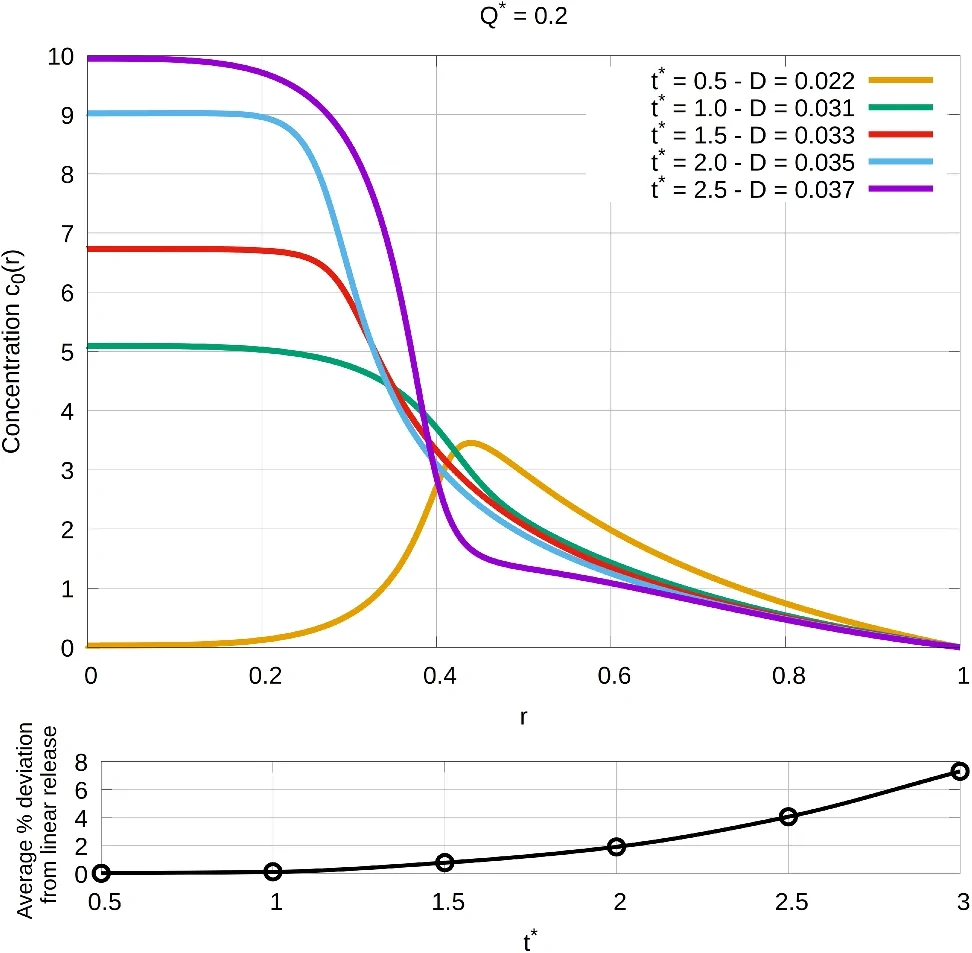
Top: Optimal initial drug concentration $c_0(r)$ with varying $t^*$ assuming constant D=0.037 and $Q^*=0.2$. As $t^*$ is increased, more drug needs to be loaded further away from the release medium so as to ‘top up’ the release rate. Bottom: Increasing $t^*$ results in greater average deviation from a constant release rate

The ability to obtain a constant release rate is limited to some time of interest $0<t<t^*$, due to the nature of diffusion problems with a finite source. Thus, it is of interest to explore how varying $t^*$ affects the optimal $c_0(r)$, assuming the same optimal D=0.037. In Fig. 7, we observe that altering $t^*$ results in a redistribution of $c_0(r)$ to maintain the constant release rate. Specifically, as we increase $t^*$, more drug is distributed further from the release medium. While the above analysis considered a fixed $Q^*$, it follows that the smaller the value of $Q^*$ desired, the lower the constant diffusion coefficient required to maintain the slower release. However, for a representative case of $P(t^*)=0.5$ (i.e. 50% release) and $Q^*=0.05-0.2$, only minor differences in the optimal $c_0(r)$ are observed (results not shown). Taken together, our results indicate that if constant release is desired, then spatially-uniform loading of drug is sub-optimal.

### Targeting a More General Release

Results of the previous section show that a constant release rate can be achieved by modulating the drug initial concentration, while selecting an appropriate constant diffusion coefficient. In this section, we look at the more general problem of how to obtain a desired drug release profile that does not possess a constant release rate. For the purposes of this study, we restrict ourselves to the consideration of diffusive drug transport. While this limits the family of release curves that may be obtained, it nonetheless allows us to attempt to optimise the FGM properties to obtain a release curve that passes through a finite number of desired ‘checkpoints.’ This represents a more realistic scenario whereby clinicians are likely less concerned with the full release curve and more interested in the percentage of drug released at some fixed time points.

Before moving to the optimisation problem, we first solve the forward problem with an assigned FGM diffusivity function $D(r)=1 +(D_{\text {min}}-1) f(r)$ (nondimensional) and choose $c_0=1$ for simplicity. This provides release curves from which checkpoints can be selected to test the ability of the optimisation procedure to recover the full curves. As test cases, we select two forms for *f*, say:

3.2 $$\begin{aligned} (1) \qquad f_1(r)= {1 \over 2} + {1 \over \pi } \arctan \left( 20 \left( {r - 0.5} \right) \right) , \end{aligned}$$

**Fig. 8:**
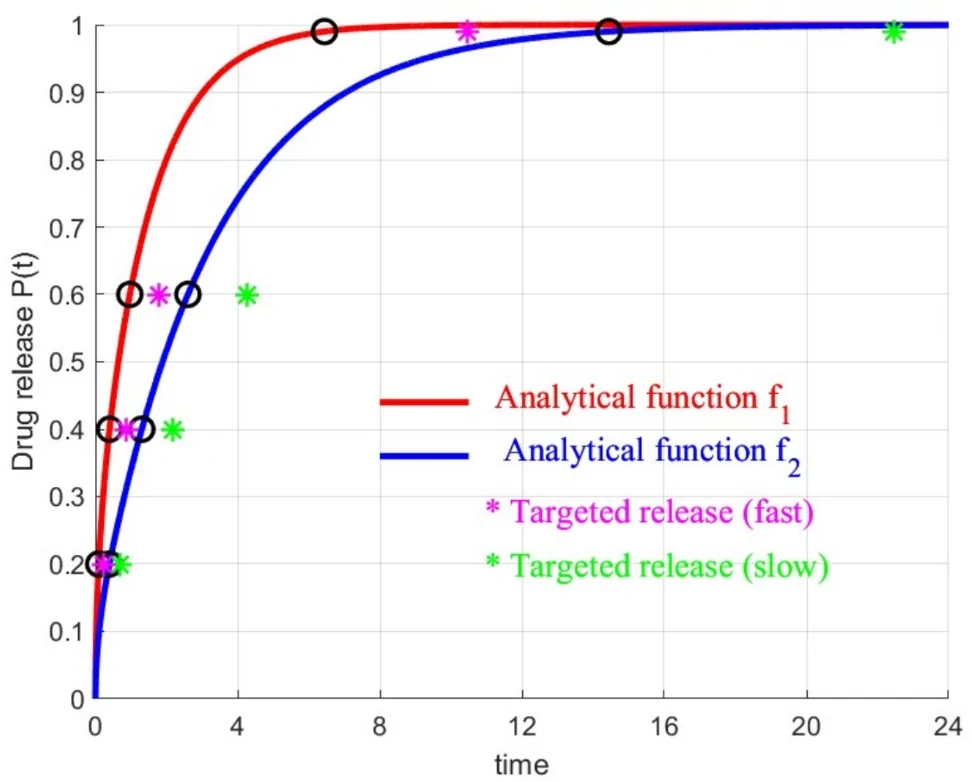
Forward simulations of drug release assuming the analytical forms of *D*(*r*) given by Eq. (3.2) (red) and Eq. (3.3) (blue), with circles indicating $20\%, 40\%, 60\%$ and 99% release. The stars correspond to arbitrary desired checkpoints, corresponding to faster (magenta) and slower (green) release, respectively, that we will attempt to recover by minimising the objective function (3.4)

3.3 $$\begin{aligned} (2) \qquad f_2(r)= {1 \over 1 + \exp (50 \,(r-0.8))}, \end{aligned}$$

corresponding to a faster (1) and slower (2) release, respectively. The functions $f_1(r)$ and $f_2(r)$ were selected as illustrative benchmark profiles rather than derived from a specific experimental material system. Both are smooth, bounded, monotonically decreasing functions that generate spatially graded diffusivity distributions through $D(r)=1 +(D_{\text {min}}-1) f(r)$. Such sigmoidal forms provide a simple way of representing functionally graded materials in which the diffusivity transitions gradually between high- and low-diffusivity regions [18]. The specific arctangent form (3.2) gives a relatively broad transition centred within the sphere and therefore produces faster release, whereas the specific logistic form (3.3) gives a sharper transition located closer to the outer region and therefore produces slower release. These two cases were chosen to provide contrasting release behaviours with which to test whether the inverse optimisation framework could recover prescribed release profiles from a limited number of target checkpoints. Figure 8 shows the generated release profiles, from which we identify 4 checkpoints corresponding to $P_1=20\%, \;P_2=40\%, P_3=60\%$ and $P_4=99\%$ release [18, 19]. Then the inverse problem is solved as in the previous section, again utilising Bézier curves, with objective function:

3.4 $$\begin{aligned} \mathfrak {G} (X_1, X_2,...,X_M)=\sqrt{\frac{\displaystyle \sum _{i=1}^{4} \left( P(t_i) - P_i \right) ^2}{4}} . \end{aligned}$$

**Fig. 9:**
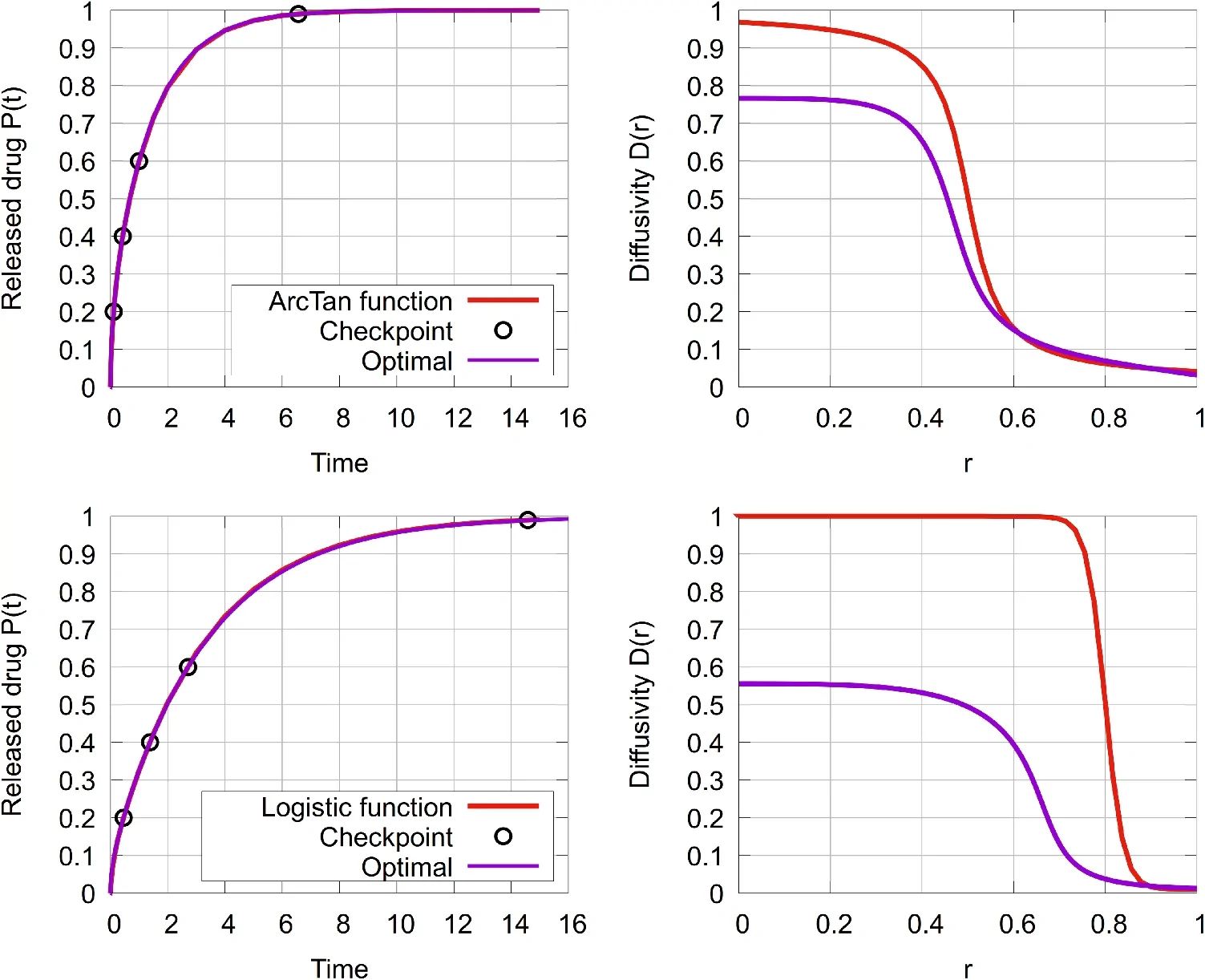
Left: Comparison between optimal release and forward simulation of drug release assuming FGM associated with Eq. (3.2) (fast release, top) and with Eq. (3.3) (slow release, bottom). Right: Comparison between *D*(*r*) obtained through the optimisation procedure utilising the checkpoints ($20\%, 40\%, 60\%$ and 99% release) (purple curve) and the analytical form of *D*(*r*) associated with Eq. (3.2) (top) and Eq. (3.3) (bottom) (red curves)

We then optimize over *D*(*r*) subject to the constraints in Table 1. Figure 9 (top left) confirms that our methodology recovers the correct release profile for the fast case. However, Fig. 9 (top right) shows a different *D*(*r*) compared with that utilized in solving the forward problem. That is, there are at least two, and indeed many *D*(*r*) that can produce the same release profile. In other words, the optimal *D*(*r*) is not unique. This is not a problem from the clinical viewpoint, provided that the correct release profile is produced. However, taking into account manufacturing considerations, one ‘optimal’ form of *D*(*r*) may be preferable over the others. Figure 9 (bottom) demonstrates that we are also able to recover the slow release profile. Similarly to the fast release case, we recover a different *D*(*r*) compared with that utilized in solving the forward problem.

**Fig. 10:**
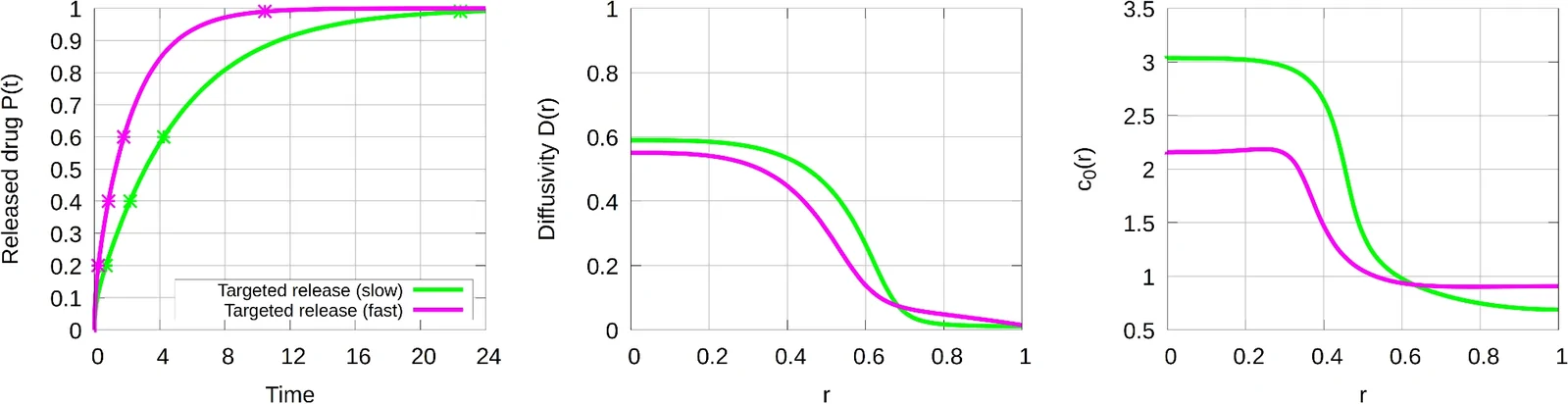
Optimal release curves (left), *D*(*r*) (centre) and $c_0(r)$ (right) associated with the arbitrary drug release checkpoints (magenta and green) defined in Fig. 8

We now proceed to perform the more general optimisation over both *D*(*r*) and $c_0(r)$ for an arbitrary set of checkpoints (see starred points in Fig. 8). We show that using our framework it is possible to find a combination of $c_0(r)$ and *D*(*r*) that recovers the desired release to an acceptable level of accuracy (Fig. 10). This demonstrates how the flexibility afforded by spatially-varying diffusivity and initial drug distribution can help to achieve more general release curves. Clearly, there are limitations in terms of the full range of release curves that can be obtained: since diffusion remains the main underlying mechanism of release, curves which exhibit temporary plateaus, changes in concavity or entirely arbitrary shapes may not be achievable.

## Discussion

The primary objective of this study was to investigate whether diffusion-controlled drug delivery systems can be rationally engineered to achieve prescribed release behaviours through spatial control (FGM) of material properties and initial drug loading. By combining a continuum-scale transport model with inverse optimisation, we have demonstrated that diffusion-controlled systems possess greater flexibility than is often assumed. In particular, the results show that near zero-order release can be achieved over a prescribed time window through appropriate spatial tailoring of the initial drug distribution, while more general release behaviours require simultaneous control of both the initial drug loading and spatial diffusivity.

One of the most significant findings is that spatial variation in diffusivity alone was insufficient to generate a constant release rate. In contrast, optimisation of the initial drug distribution proved capable of producing near zero-order release when combined with a suitable constant diffusivity. This result has important implications for the design of diffusion-controlled drug delivery systems. Considerable effort is often devoted to modifying material properties in order to influence release kinetics. However, our results suggest that, for the specific objective of achieving constant release, engineering the spatial distribution of drug within the device may be a more effective design strategy than engineering spatially varying transport properties. Furthermore, because diffusivity primarily controls the overall timescale of release, the role of material selection may be viewed as determining the duration over which approximately constant release can be maintained, while the drug loading profile determines the shape of the release curve.

These findings are also relevant from a manufacturing perspective. A key question is how the optimised diffusivity and drug loading profiles identified by the model might be realised experimentally. In the present framework, spatially varying diffusivity is represented through the function *D*(*r*). In practice, such diffusivity gradients could arise from controlled spatial variations in polymer composition, porosity, crosslink density, crystallinity, and other microstructural features known to influence drug transport [29]. These types of gradients are increasingly accessible through advances in functionally graded materials and pharmaceutical manufacturing technologies [30, 31]. Similarly, the non-uniform initial drug distributions predicted by the optimisation framework may be achievable using fabrication strategies such as sequential coating processes, solvent-based deposition methods, and additive manufacturing techniques [32]. Importantly, our results suggest that tailoring the spatial drug distribution may in some cases be more effective, and potentially more straightforward, than achieving precise spatial control of diffusivity. Consequently, the optimised functions *D*(*r*) and $c_0(r)$ should not be interpreted as prescribing a unique material formulation. Rather, they define target transport-property and drug-loading distributions that can guide material selection and device fabrication. Viewed in this way, the optimisation framework may serve as a computational design tool capable of identifying promising material architectures prior to fabrication, thereby reducing reliance on costly experimental trial-and-error approaches.

From a clinical perspective, the ability to design drug delivery systems around a desired release profile rather than accepting the profile dictated by a given single or multi-layer material system may offer substantial advantages. Different therapeutic applications often require markedly different dosing strategies. In some cases, maintaining a near constant drug release rate is desirable to sustain concentrations within a therapeutic window while minimising fluctuations associated with toxicity or loss of efficacy. In other situations, delayed, accelerated, or application-specific release profiles may be preferred. The inverse-design framework developed here provides a route towards tailoring device properties to achieve specific therapeutic objectives. While the present study does not explicitly consider pharmacokinetics or pharmacodynamics, the methodology could readily be extended to incorporate such models, enabling optimisation against clinically relevant concentration-time profiles rather than release characteristics alone.

The results obtained for the more general optimisation problem further demonstrate the flexibility afforded by combining spatially varying diffusivity with non-uniform initial drug loading. We showed that prescribed release checkpoints could be reproduced to a high degree of accuracy using suitably engineered functionally graded materials. Interestingly, the optimisation procedure often identified diffusivity distributions that differed from those used to generate the target release profiles. This observation highlights an important feature of the inverse problem, namely the potential non-uniqueness of optimal solutions. Multiple combinations of material properties and loading profiles may produce similar release behaviour. From a practical perspective, this non-uniqueness may be advantageous, as it provides manufacturers with flexibility to select designs based on considerations such as manufacturability, robustness, cost, or regulatory constraints while still achieving the desired therapeutic outcome.

Several limitations of the present study should be acknowledged. First, the model assumes diffusion-controlled transport under perfect sink conditions and neglects additional mechanisms such as polymer degradation, swelling, erosion, dissolution, and drug-polymer interactions. These effects are known to influence release kinetics in many practical systems and would expand the range of attainable release profiles. Second, the study focuses on demonstrating the optimisation methodology and does not include direct experimental validation. While the purpose of the present study is to establish a computational framework for the inverse design of diffusion-controlled drug delivery systems rather than to model a specific experimental formulation, experimental validation of the optimisation framework represents an important next step. Such studies would involve manufacturing systems with prescribed spatial drug loading and/or diffusivity distributions and comparing measured release kinetics against model predictions. Advances in additive manufacturing, microfluidic fabrication, and functionally graded materials may provide suitable experimental platforms for testing the design principles identified in this work.

An important avenue for future work is the extension of the framework to more sophisticated release behaviours, including multi-stage, and time-programmed drug release. Although purely diffusive systems are inherently constrained in the release profiles they can generate, incorporation of additional mechanisms such as degradation, swelling, moving interfaces, or externally triggered changes in transport properties would substantially enlarge the space of attainable release behaviours. Coupling such models with the optimisation framework developed here could provide a powerful platform for the rational design of next-generation drug delivery systems capable of delivering highly tailored therapeutic regimens.

Overall, the present work demonstrates how mathematical modelling and optimisation can be used not only to analyse existing drug delivery systems but also to guide their design. By framing drug delivery design as an inverse problem, the proposed methodology offers a systematic route for identifying material and loading configurations capable of achieving prescribed release objectives, thereby supporting the development of more effective and application-specific drug delivery technologies.

## Conclusions

Diffusion based DDS, while ubiquitous, have limitations in terms of their ability to maintain zero-order release and release that deviates from constant diffusive properties. In this paper, we have demonstrated that, to obtain constant release rate over a desired time period, it is sufficient to design a system with a spatially-varying initial drug loading. Incorporating an additional spatially-varying diffusivity (FGM) confers no advantage in the pursuit of zero order release. However, since diffusivity controls the time scale of the release, the magnitude of diffusivity is important in determining the time period over which zero-order release may be maintained. When we consider more arbitrary drug release, motivated by a desire to achieve a set of drug release checkpoints, we find that FGM, in conjunction with spatially-varying initial drug loading, can be used to tune the desired release profile. Together, our results motivate the design of FGM for DDS that enable non-uniform drug loading and spatially-varying diffusion properties.

## Data Availability

All data supporting this study are provided within the main text.
